# Epigenetic biomarkers for animal welfare monitoring

**DOI:** 10.3389/fvets.2022.1107843

**Published:** 2023-01-11

**Authors:** Rose Whelan, Sina Tönges, Florian Böhl, Frank Lyko

**Affiliations:** ^1^Creavis, Evonik Operations GmbH, Hanau, Germany; ^2^Innovation Management, German Cancer Research Center, Heidelberg, Germany; ^3^Division of Epigenetics, DKFZ-ZMBH Alliance, German Cancer Research Center, Heidelberg, Germany

**Keywords:** animal welfare, origin tracing, epigenetics, DNA methylation, biomarker

## Abstract

Biomarkers for holistic animal welfare monitoring represent a considerable unmet need in veterinary medicine. Epigenetic modifications, like DNA methylation, provide important information about cellular states and environments, which makes them highly attractive for biomarker development. Up until now, much of the corresponding research has been focused on human cancers. However, the increasing availability of animal genomes and epigenomes has greatly improved our capacity for epigenetic biomarker development. In this review, we provide an overview about animal DNA methylation patterns and the technologies that enable the analysis of these patterns. We also describe the key frameworks for compound DNA methylation biomarkers, DNA methylation clocks and environment-specific DNA methylation signatures, that allow complex, context-dependent readouts about animal health and disease. Finally, we provide practical examples for how these biomarkers could be applied for health and environmental exposure monitoring, two key aspects of animal welfare assessments. Taken together, our article provides an overview about the molecular and biological foundations for the development of epigenetic biomarkers in veterinary science and their application potential in animal welfare monitoring.

## Introduction

Due to growing consumer concerns for animal welfare, the definitions and assessment tools for measuring animal welfare are continually being investigated and improved (1). To date, for most welfare assessments, resource-based measures (e.g., stocking density or drinker space) and animal-based measures (e.g., levels of specific types of lameness, disease, or stress) are used in conjunction to try to gain a holistic view on welfare. Multiple measurements must be taken from the time the animals arrive on farm until they are slaughtered, and most measurements can only assess welfare at the exact timepoint of testing. Tools for a more holistic monitoring of the veterinary health status that captures the entirety of the animals' lifespan are currently lacking. This review proposes to develop epigenetic biomarkers to provide novel solutions for the analysis of animal welfare and other attributes of interest for the animal agriculture industry.

Epigenetic mechanisms allow the context-dependent expression of genetic information. This is exemplified by the mechanisms that underpin cellular differentiation: while the many cell types of an individual organism largely contain the same DNA sequence, they can have very different phenotypes with very different functions. Epigenetic mechanisms canalize specific cell fates and mediate their plasticity by establishing, maintaining and modulating cell type-specific gene expression patterns. These features establish epigenetics as an appealing concept for many scientific fields, particularly at the interface between the genome and the environment (2, 3).

On the mechanistic level, epigenetic regulation is usually linked to DNA methylation, histone modifications and small RNAs. Among these, DNA methylation represents the longest known and best analyzed epigenetic modification. About 4–5% of the cytosines in the human genome are present as 5-methylcytosine (4), which has also been termed “the fifth base” of the human genome. Notably, DNA methylation represents the only known epigenetic modification with a clearly defined mechanism for mitotic heritability, which ensures the stable maintenance of cell-fate decisions (5, 6). These decisions are re-set by epigenetic erasure mechanisms during germline and early embryonic development (7), thus preventing transgenerational inheritance of DNA methylation patterns (8). Nevertheless, the dynamics of DNA methylation establish the modification as an attractive biomarker for health and disease. DNA methylation biomarkers have been successfully established for cancer diagnosis (9), as well as human age prediction and life expectancy (10). However, considering the wide evolutionary conservation of the modification, DNA methylation biomarkers remain underexplored in other species.

For a long time, DNA methylation research has been focused on human, mouse and a few other models. However, the availability of next-generation sequencing technologies has enabled epigenome mapping for any species with a complete genome sequence (11). This has reinforced longstanding research interests in evolutionary and environmental epigenetics. While evolutionary analyses have revealed the considerable diversity of animal epigenomes, they were largely focused on evolutionarily well-characterized groups, such as insects (12). However, we still lack detailed knowledge about the epigenomes of many species that are relevant for veterinary medicine, such as agricultural livestock. While the broad importance of epigenetics for livestock management has been reviewed elsewhere (13), this article focuses on the potential of DNA methylation patterns for the development of animal welfare biomarkers.

## DNA methylation patterning in animals

Cytosine-5 DNA methylation has been highly conserved in eukaryotic evolution (14, 15). The large majority of methylated cytosines is found in the context of CpG dinucleotides, which ensures the post-mitotic heritability of the modification from the parental DNA strand to the daughter strand through a dedicated maintenance mechanism (5, 6). However, not all CpG dinucleotides are methylated in a given genome and the epigenetic information is encoded by the distribution pattern of methylation marks, also called the DNA methylation “landscape” (16–18). These DNA methylation landscapes can be viewed as barcodes that encode the context-dependent information of a specific genome.

The key forces that shape DNA methylation landscapes are the antagonistic activities of DNA methyltransferases and demethylases. DNA methyltransferases are encoded by the DNMT family of genes that use S-adenosylmethionine as a methyl-group donor (19). Demethylases are encoded by the TET family of dioxygenases that oxidize 5-methylcytosine to promote the reversal of methylation marks (20). On the molecular level, TET-mediated oxidation antagonizes the methylation activity of DNMTs, thus leaving certain regions of the genome unmethylated (21). This dynamic equilibrium between DNMTs and TETs provides an attractive mechanistic explanation for the methylation dynamics that have been observed at mammalian gene regulatory sequences.

On the molecular level, DNA methylation is generally considered to restrict transcription factor binding, thereby affecting gene expression (22). In agreement with this notion, many transcription factors have been shown to preferentially bind unmethylated target sequences over methylated target sequences (23). However, other transcription factors, including the homeodomain family, showed a preference for methylated sequences, which was explained by direct hydrophobic interactions with the methyl-group of 5-methylcytosine (23). These findings illustrate the complexities of the interaction between DNA methylation and transcription factors and how they can depend on molecular details.

**Figure 1:**
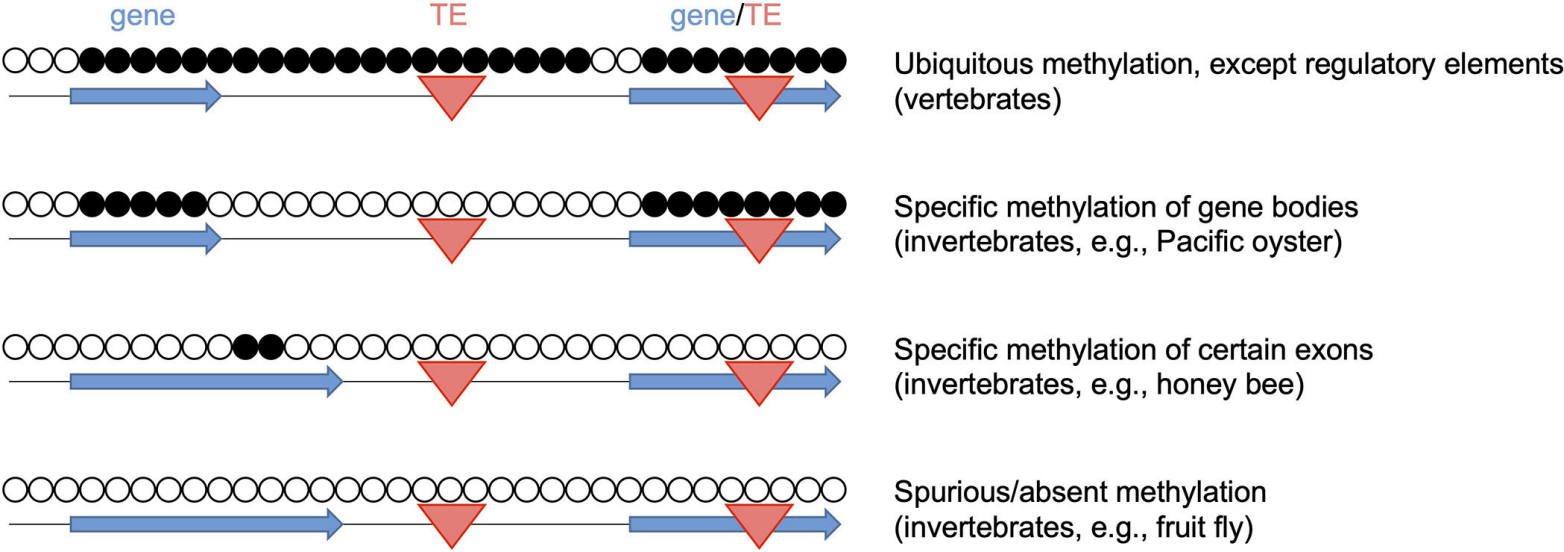
Diversity of DNA methylation patterns across the animal kingdom. Open and closed circles represent unmethylated and methylated CpGs, respectively. Blue horizontal arrows: gene bodies, red triangles: transposable elements (TE). The representative methylomes are the Pacific oyster (24), the honey bee (25) and the fruit fly (26).

The evolutionary diversity of animal DNA methylation patterns (Figure 1) contradicts the widespread assumption that key features of human or mammalian methylomes are conserved. For example, the high methylation density of mammalian genomes becomes strongly reduced at the base of the vertebrate clade (27). Also, DNA methylation density has been shown to be increased and decreased repeatedly during insect evolution, suggesting that methylation patterning is highly diverse across the animal kingdom (12, 28). This is also reflected by known livestock methylomes. For example, the methylation landscape of pigs has been shown to be similar to the paradigmatic human and mouse methylomes, with high overall methylation, but low promoter methylation (29). The methylomes of non-mammalian vertebrate livestock, such as chicken, also appear similar, but show some notable differences, including reduced global methylation levels, which might be attributed to the non-conservation of a methylation cofactor (30). Finally, invertebrate/aquatic livestock, such as the Pacific oyster (24), often show strongly different landscapes that are characterized by more restricted, gene body-specific methylation.

It should be noted that some insect lineages, such as dipteran flies, completely lack DNA methylation, while retaining the full capacity to execute cellular differentiation programs (26). These findings raise important questions about the conservation of DNA methylation functions. Indeed, the conserved functional roles of DNA methylation in animals remain to be fully understood. In mammals, DNA methylation is essential for development and cell-fate specification (31). This function appears to be conserved to some degree, as tissue-specific methylation patterns have also been described in other vertebrates, including chicken and salmon (30, 32). However, DNA methylation patterns have been described as tissue-invariant in several invertebrate species, including tunicates, crayfish and honeybees (33–35) and the function(s) of DNA methylation in organisms with invariant methylomes remain(s) to be established. The mechanisms that drive the evolutionary diversity of methylation patterns might provide valuable information in this context, but remain to be fully understood. Notably, however, several studies have suggested a close link between transposable elements and the diversification of DNA methylation patterns. For example, insertions of hypomethylated transposable elements have been associated with a reduction of global DNA methylation levels during arthropod evolution (28). On the other hand, methylation of transposable elements can result in increased genome methylation levels (36). Finally, the loss of methylated transposable elements can cause a complete loss of DNA methylation, as observed in some insect lineages (28). These findings make it plausible to assume a conserved functional role of DNA methylation in the control of transposable elements. However, compelling evidence in support of this function presently remains limited to mice (37, 38).

## Technologies for DNA methylation analysis

Chemically, DNA methylation is defined by the strong covalent carbon-to-carbon bond that connects the methyl-group to the cytosine residue. As such, DNA methylation has several features that distinguish it from other epigenetic modifications, such as histone modifications and small RNAs. For example, DNA methylation is a direct chemical modification of DNA, which can be analyzed without any requirements for the preservation of associated molecules and structures. Also, it can be detected at single-base resolution, and thus achieves the highest possible level of resolution for an epigenetic mark. Furthermore, the stability of the modification greatly facilitates sample preservation and limits artifacts that are due to sample storage. In principle, DNA methylation is as stable as DNA itself and thus represents an excellent substrate for analytical detection.

The methodological challenges for the detection of DNA methylation patterns lie in the relatively limited biochemical impact of the methyl-group. For example, methyl-groups have a negligible effect on the hybridization of nucleic acids, thus precluding the use of many traditional molecular detection methods. Methylation-sensitive restriction enzymes were valuable tools during the early years of molecular epigenetics research (39), but they limited DNA methylation analyses to a small set of restriction enzyme target sites. Another widely used tool for DNA methylation analysis is based on antibodies that have a certain amount of specificity for methylated DNA. These antibodies form the backbone of immunoprecipitation-based methods, such as me-DIP (39). However, it has been shown that 50–99% of regions that are enriched by these protocols are caused by the intrinsic affinity of IgG to short unmodified repeats and thus represent artifacts that are unrelated to DNA methylation (40).

The arguably most robust methods for DNA methylation analysis are based on the ability of sodium bisulfite to induce a methylation-dependent polymorphism into the DNA sequence (39): sodium bisulfite deaminates unmethylated cytosines to uracils, while methylated cytosines (and its oxidated derivatives, such as 5-hydroxymethylcytosine) are protected against deamination. A subsequent PCR reaction converts uracils (derived from unmethylated cytosines) to thymines, while modified cytosines remain as cytosines. Methylation calling is then based on bisulfite-induced genetic polymorphisms and allows the quantitative mapping of methylation marks within the PCR-amplified DNA fragments at single-base resolution. When this workflow is integrated into whole-genome sequencing protocols, it allows the establishment of genome-wide methylation maps. The corresponding method is called whole-genome bisulfite sequencing (WGBS) and represents the gold standard for DNA methylation analysis (41).

Because WGBS datasets contain millions of independent and variable data points, their analysis is challenging and time-consuming. In addition, WGBS requires considerable (>20X) sequencing coverage to achieve reasonable robustness, which can cause high costs. Finally, it has been shown that 70–80% of WGBS sequencing reads from human samples never change their methylation, suggesting that a more focused approach that is based on dynamically methylated subgenomes is more effective (42). One widely used approach for subgenome bisulfite sequencing is reduced representation bisulfite sequencing (RRBS) (43), which uses digestion with methylation-sensitive restriction enzymes to enrich CpG-dense regions of the genome. Similarly, it is possible to capture specific genomic regions of interest with biotinylated RNA oligonucleotide “baits” (44) before analyzing them by bisulfite sequencing. Both methods usually produce higher sequencing coverage and thus allow more robust methylation calling at lower costs. However, RRBS shows a strong bias for CpG-dense regions, while capture-based bisulfite sequencing requires a considerable investment in the design and production of the capture beads.

Bisulfite-induced, methylation-dependent polymorphisms can also be detected on array-based platforms. Arrays have played a major role in the analysis of nucleic acids, as they are often more streamlined and cost-effective than sequencing-based technologies. Arrayed primer extension (45) has provided a particularly robust solution for the detection and quantification of methylation marks and currently allows the analysis of more than 850.000 CpGs in the human genome (46). Methylation arrays have played an instrumental role in analyzing and characterizing the healthy and diseased human methylome. It will be critically important to develop similar platforms for relevant animal genomes in order to fully realize the potential of epigenetic biomarkers for the analysis of animal health and disease.

While the robustness of bisulfite-dependent technologies has greatly advanced our understanding of animal methylomes, studies need to be designed carefully, in order to avoid artifacts and confounding factors (11). For example, animal populations often show substantial genetic heterogeneity, and genetic polymorphisms at cytosine residues can result in artifactual loss-of-methylation calls. It is therefore important to remove all polymorphic sites from the methylation data analysis. Furthermore, DNA samples should be carefully prepared from specific tissues to minimize confounding effects that stem from changes in the cell-type composition of the samples. Lastly, it is important to avoid over-interpretation of very small methylation differences, as they are unlikely to have a biological impact.

## DNA methylation biomarkers for veterinary health monitoring

Over the past years, molecular biomarkers have developed into a key tool for modern agriculture, as they allow the monitoring of large groups of animals and provide objective quality assurance (47). In this context, epigenetic biomarkers are considered particularly promising, because of their ability to integrate multi-dimensional, context-dependent information. Of note, the increasing availability of methylation data from livestock already allows the development of complex DNA methylation biomarkers (48). An interesting example is the development of DNA methylation clocks for ruminant livestock, based on a pan-mammalian DNA methylation array that covers roughly 40,000 conserved CpGs (49). DNA methylation clocks are compound DNA methylation biomarkers that can accurately predict chronological and biological age of a sampled specimen (10). This information could greatly aid herd management and breeding decisions for animal livestock management (48). A DNA methylation clock has also been established for broiler chickens, based on WGBS datasets (30). Interestingly, the chicken clock showed accelerated aging upon the experimental induction of systemic inflammation, suggesting that it is capable of predicting broiler health and performance (30). Methylation age acceleration has also been associated with adverse health conditions in humans and in mice (10) and represents a novel and highly innovative biomarker candidate for livestock management.

Additional, conceptually novel epigenetic biomarkers can be developed through the influence of environmental factors on DNA methylation patterns. In animals, this topic is often discussed in the context of invasive species, as they have the capacity to rapidly adapt to different environments (50). This rapid adaptation cannot be explained by the selection of genetic variants, which is a comparably slow process that requires multiple generations. For example, DNA hypomethylation in a recently established colony of the pygmy mussel (*Xenostrobus secures*) was interpreted to facilitate phenotypic adaptation (51). Another interesting study on farmed coho salmon (*Oncorhynchus kisutch*) associated the lower fitness of hatchery-reared salmon with DNA methylation patterning. The results showed a significant number of methylation differences between hatchery-reared animals and wild animals from the same river (52), suggesting a role of DNA methylation in environment-specific adaptation.

Environment-specific DNA methylation signatures were also observed in marbled crayfish (*Procambarus virginalis*), which is a widely distributed, invasive freshwater crayfish. Due to their very young species age of about 30 years, the animals have maintained a largely monoclonal genome with negligible genetic variation (53). Using WGBS and subsequent subgenome methylation analysis, stable environment-specific methylation signatures could be observed (54). While this pilot study was greatly aided by the genetic homogeneity of marbled crayfish, a follow-up analysis also identified location-specific DNA methylation signatures in traditional aquatic and terrestrial livestock, such as shrimp, salmon and chicken (Venkatesh et al., submitted)^1^. These findings illustrate how the environment leaves a highly specific fingerprint on livestock genomes, which is a complex DNA methylation signature reflecting the vast diversity of environmental factors. The decoding of these fingerprints represents an attractive approach for developing a tampering-resistant origin tracing technology for meat and other animal products (see text footnote 1).

**Figure 2:**
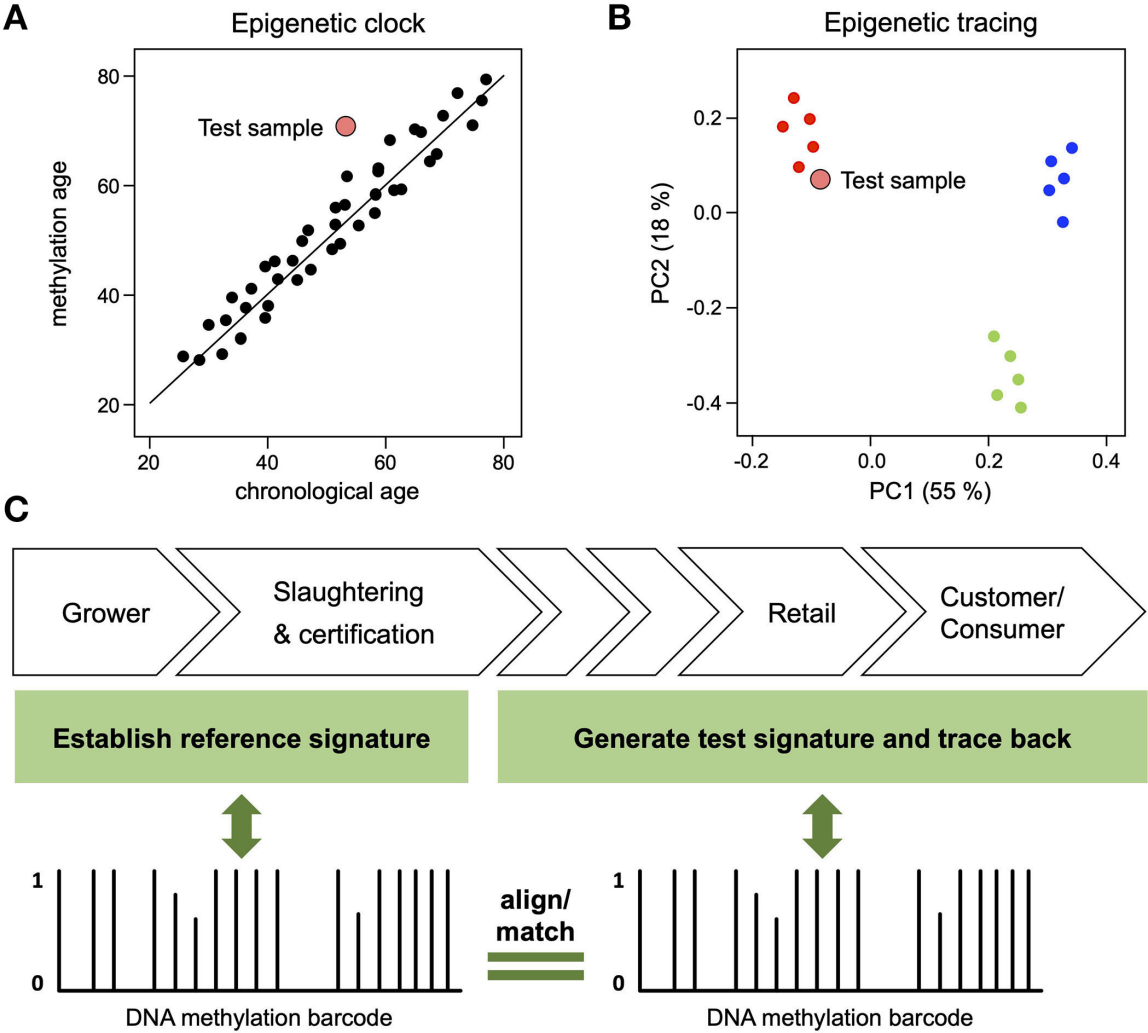
Compound DNA methylation biomarkers for animal welfare assessments. **(A)** DNA methylation analysis of a test sample (large red dot) reveals a methylation age that is higher than its chronological age, indicating that the corresponding individual was exposed to unhealthy conditions. **(B)** DNA methylation analysis of a test sample (large red dot) reveals a pattern that is similar to the reference samples provided by one specific farm (small red dots), thus pinpointing its origin from a source with high welfare standards. **(C)** Illustration of epigenetic tracing by environmental fingerprinting. Meat samples are collected on farms and/or at slaughterhouses for the establishment of reference DNA methylation signatures (“barcodes”). Test signatures can be generated from any point along the value chain and then be aligned with reference signatures. Test signatures matching a specific reference signature confirm that the animals were reared in the specific reference farm(s). Conversely, test signatures not aligning with a reference signature indicate that the animal was reared outside of the farms represented by the reference profiles.

Both DNA methylation-based origin traces and clocks represent compound biomarkers that are defined by context-dependent methylation variability. While the methylation of clock marks varies with age, the methylation of environmental marks varies with environmental factors. Importantly, both frameworks provide important information for animal welfare assessments: if a methylation clock analysis shows a higher methylation age than chronological age (Figure 2A), this indicates that the corresponding animal was exposed to unhealthy conditions (e.g., disease, poor nutrition, stress, poor hygiene etc.), resulting in age acceleration (10). Similarly, if an environmental trace shows a high similarity to specific references (Figure 2B), this indicates similar environmental parameters and a shared origin. Their capacity to integrate multi-dimensional biological variables distinguishes these compound epigenetic biomarkers from more conventional, genetic biomarkers that are currently used to monitor animal breeding and rearing.

While the mechanisms that shape environment-specific epigenetic signatures remain to be fully understood, several observations have been made that provide first cues. For example, it has been shown that environmental signatures in chicken are associated with genes involved in transcriptional regulation and cellular differentiation (see text footnote 1), consistent with the known function of DNA methylation in mammals (31). It has also been shown that the chicken methylome is dynamically methylated at transcription factor binding sites (30), which provides an explanation for how methylation variability could impact gene expression. Finally, a moderate, but significant enrichment of transposable elements in the regions surrounding differentially methylated CpGs has been described in chicken (see text footnote 1) and in marbled crayfish (54), which is consistent with the known association between transposable elements and epigenetic variation (55).

## Application potential of DNA methylation biomarkers in animal welfare assessment

The needs for biomarker-based analytics in veterinary medicine are diverse. As mentioned above, methylation clocks may be used to identify reduced health status in real time during the design of breeding programs or the rearing of livestock on farm. However, the uses for the clock extend beyond the age estimation of the animal. As incomes and education levels rise, consumers have been shown to take a greater interest in the quality of food they are eating and are increasingly willing to pay premiums for meat products that align with their values, including animal welfare (56). Currently, this is mostly assessed *via* audits on farm with visual assessments of housing conditions and animal physiology on a macroscopic level. However, the are no internationally agreed standards or assessments of animal welfare to include in audits, with some aspects designed based on human perception rather than data-driven assessments of welfare. This can create confusion for import-export markets and a lack of trust in welfare labels. DNA-methylation clocks for agriculturally relevant species, like broiler chickens (30), could provide a method by which the comprehensive effect of all the experienced health states can be conclusively assessed. The use of DNA-methylation clocks for welfare assessments would not be replacing existing technologies, but rather providing the first analytics for the overall experienced welfare of animals.

Environment-specific DNA methylation signatures in livestock, could likewise provide analytics to monitor the breeding and rearing in health-promoting environments. In this regard, consumers have been shown to universally value transparency regarding the source from which their food is derived (56). Through analysis of the methylation patterns of animals from the same species, but reared in different regions, epigenetic reference profiles can be obtained, reflecting the unique combination of environmental factors for each region. When the methylation patterns of the relevant CpG sites are analyzed in an unknown sample the similarity to a reference profile can confirm the animal was reared in a specific (e.g., health-promoting) environment, while conversely a significant difference to a reference profile can confirm an animal was not reared in that environment (Figure 2C).

Environment-specific DNA methylation signatures could also be used to assess specific meat quality certifications relating to how an animal was reared. For example, animal products can be certified and labeled with many distinct attributes including antibiotic-free, GMO-free, raised without hormones, halal, kosher, organic, free range, pasture raised, grass fed, grain fed, etc. Establishing DNA methylation profiles to distinguish these specific characteristics through analysis of meat products would provide transparency and assurance to consumers. To date, many of these attributes cannot be assessed with analytics and are again reliant on audits. Where analytics do exist, they may be lacking in accuracy. For example, antibiotic testing currently involves chemical analysis to find residues of antibiotics present in the meat, milk or eggs of animals (57). However, most antibiotics used for growth promotion in livestock are added to the feed or water of the animals and most of the antibiotic classes used are poorly absorbed in the gastrointestinal system. Therefore, residue testing in meat, milk or eggs may not capture usage of all antibiotic classes, particularly after the withdrawal period (where the animal is no longer given the antibiotics) or where residues are below detection limits for traditional chemical analytic methods. As DNA methylation effects may persist after the cessation of treatment, methylation signatures could be used to assess antibiotic usage at any age of animal rearing regardless of whether residues are still present at sufficient concentrations in the meat tested after slaughter.

DNA methylation signatures may thus provide a solution for the unforgeable assessment of multiple aspects of quality and certification from a single small sample of meat. The exceptional stability of DNA methylation patterns in post-mortem tissues provides an important foundation for this approach (58). Methylation-based testing would not only improve the assurance from current auditing systems but would reduce the overall number of samples and analytics required to assess all the consumer-interest aspects of meat. However, sequencing technologies while highly informative in the development phases, are also costly, time-consuming, and computationally intensive. This is not suitable for meat analytics where profit margins are low and results are required within days of sampling. However, array-based technologies have already proven their cost-effectiveness for livestock breeding (59, 60) and have the capacity to provide the high throughput and robustness required to determine quantitative methylation profiles on livestock genomes. The development of such arrays is currently underway, but limited by its restriction to evolutionary conserved methylation marks (49, 61). A methylation-based array that contains probes specific for multiple species, with methylation sites that are of interest for distinct assessment profiles (e.g., countries of origin, antibiotic usage, slaughter methods etc.) would further improve the flexibility of the technology for industrial analytic labs assessing many types of meat products, as well as research groups working with many species. Such DNA methylation-based arrays could solve many analytic needs currently lacking in the animal agriculture industry, making formerly inaccessible technology affordable and accessible to a wide range of potential users throughout the industry.

## Conclusions

Research over the past few years has shown that DNA methylation patterns can be highly diverse across the animal kingdom. The increasing availability of robust technologies for methylation mapping at single-base resolution has unlocked the potential of epigenetic biomarkers for animal welfare assessment. Notably, it has already been demonstrated that it is possible to develop compound biomarkers for animal livestock that integrate multi-dimensional, context-dependent information. Potential application fields include the quantitative assessment of animal welfare through DNA methylation clocks and tamper-resistant origin tracing of meat products by environment-dependent DNA methylation signatures. To fully realize their potential, these biomarkers will require robust technology platforms. Customized methylation arrays (61) represent an attractive option for this purpose, as they are cost-efficient, reliable and easy to analyze.

## Author contributions

All authors contributed to the writing of the manuscript, read, and approved the final manuscript.

## Footnotes

^1^Venkatesh G, Tönges S, Hanna K, Ng YL, Whelan R, Andriantsoa R, et al. Context-dependent DNA methylation signatures in animal livestock (submitted).
